# Clinicians Who Practice Primarily in Nursing Homes and the Quality of Care for Residents With Alzheimer Disease and Related Dementias

**DOI:** 10.1001/jamahealthforum.2025.2465

**Published:** 2025-08-15

**Authors:** Hyunkyung Yun, Mark Aaron Unruh, Yuting Qian, Yongkang Zhang, Hye-Young Jung

**Affiliations:** 1 Department of Health Services, Policy & Practice, School of Public Health, Brown University, Providence, Rhode Island; 2 Department of Population Health Sciences, Joan and Sanford I. Weill College of Medicine, Cornell University, New York, New York; 3 Department of Health Policy and Management, Yale School of Public Health, New Haven, Connecticut

## Abstract

**Importance:**

The number of physicians and advanced practitioners (APs) whose care is concentrated in nursing homes (often referred to as nursing home or skilled nursing facility specialists [SNFists]) has increased rapidly. Therefore, whether these clinicians provide better care is important.

**Objective:**

To examine the association between SNFist care and outcomes of long-stay nursing home (NH) residents with Alzheimer disease and related dementias (ADRD).

**Design, Setting, and Participants:**

In this retrospective cohort study of 417 378 residents with ADRD in US NHs, claims for a 20% national sample of Medicare fee-for-service beneficiaries between 2013 and 2019 were analyzed. Adjusted estimates were based on a machine learning approach that incorporated a doubly robust procedure using a generalized estimating equation with inverse probability treatment weighting. Three secondary analyses were conducted: (1) stratified analyses for physicians and APs, (2) inclusion of physicians of any specialty and APs, and (3) use of proxy outcomes for in-place deaths. Data were analyzed from June 1, 2024, to May 3, 2025.

**Intervention:**

Receipt of care from a SNFist; SNFists included generalist physicians and APs.

**Main Outcomes and Measures:**

Hospitalizations and emergency department (ED) visits for ambulatory care–sensitive (ACS) conditions. Death without an ACS hospitalization and death without any hospitalization were used in secondary analyses.

**Results:**

Of the total 417 378 residents, 242 540 received care from SNFists (mean [SD] age, 83.5 [8.7] years), and 174 838 never received care from SNFists (mean [SD] age, 84.8 [8.5] years). Compared with the residents who never received care from SNFists, the residents who received care from SNFists were more likely to be Black (12.6% vs 9.4%; *P* < .001), dually eligible (77.5% vs 73.1%; *P* < .001), and have more chronic conditions (eg, anemia, 60.9% vs 57.6%). Compared with non-SNFist clinicians, the SNFist clinicians were more likely to be female (physicians, 37.1% vs 23.3%; APs, 88.1% vs 85.1%), practice at more facilities (mean [SD] number of facilities, 9.4 [8.7] for SNFist physicians vs 6.4 [6.1] for non-SNFist physicians; 8.6 [8.1] for SNFist APs vs 7.1 [6.8] for non-SNFist APs), and less likely to practice in rural areas (physicians, 9.3% vs 25.4%; APs, 8.1% vs 20.2%). In adjusted analyses, receiving care from a SNFist vs non-SNFist was associated with 7% lower odds of an ACS hospitalization (odds ratio [OR], 0.93; 95% CI, 0.90-0.96) and 7% lower odds of an ACS ED visit (OR, 0.93; 95% CI, 0.90-0.96). In stratified analyses, receiving care from a SNFist physician vs a non-SNFist physician was associated with 13% lower odds (OR, 0.87; 95% CI, 0.83-0.90) of an ACS hospitalization and 7% lowers odds of an ACS ED visit (OR, 0.93, 95% CI, 0.88-0.97); comparisons of SNFist APs vs non-SNFist APs were not statistically significant. Estimates from the analysis including physicians of any specialty and APs were consistent with the primary results. SNFist care was associated with increased odds of in-place death.

**Conclusions and Relevance:**

Findings of this cohort study suggest that the use of SNFists by NHs may enhance the quality of care for residents with ADRD.

## Key Points

**Question:**

Do physicians and advanced practitioners who practice primarily in nursing homes, often referred to as nursing home or skilled nursing facility specialists (SNFists), provide better care for residents with Alzheimer disease and related dementias?

**Findings:**

In this cohort study, receipt of care from a SNFist vs a non-SNFist was associated with a 7% decrease in the odds of a hospitalization and a 7% decrease in the odds of an emergency department visit for an ambulatory care–sensitive condition among residents with dementia.

**Meaning:**

These findings suggest SNFists may provide higher-quality care for residents with dementia compared with other clinicians.

## Teaser

This cohort study examines the association of nursing home or skilled nursing facility specialist care and outcomes of long-stay nursing home residents with Alzheimer disease and related dementias (ADRD).

## Introduction

The number of clinicians who provide care almost exclusively in nursing homes (NHs), often referred to as NH or skilled nursing facility specialists (SNFists), increased more than 57% from 2008 to 2017.^1^ Focused practice on NH care by clinicians has been proposed as a way to improve the quality of resident care.^2^ Previous research has examined the relationship between use of SNFists and quality of postacute care, burdensome transfers of NH residents at the end of life, and NH Care Compare ratings.^3,4,5^ Despite the increasing use of SNFists and the growing evidence of their impact on quality improvements in NHs, little is known about their association with the quality of long-term care for residents.^6^

Alzheimer disease and related dementias (ADRD) are prevalent among NH residents, with estimates ranging from 48% to 87%.^7,8^ NH-focused practice by clinicians may improve the quality of care for these residents, as SNFists are likely to be more familiar than other clinicians with the behavioral and psychiatric symptoms that residents with ADRD often experience.^9^ Additionally, residents with ADRD have complex clinical needs and may benefit from the specialized, coordinated care provided by SNFists who work directly with care teams in the NH.^10,11,12,13^ Conversely, strategies promoting NH-focused care may increase fragmentation in care by reducing continuity with primary care clinicians in the community, and clinicians who focus on NH care may lack familiarity with specialists for referrals.^2^ In this national cohort study, we examined the association between the receipt of care by SNFists and the quality of care for long-stay residents with ADRD based on hospitalizations and emergency department (ED) visits for ambulatory care–sensitive (ACS) conditions.

## Methods

This cohort study was approved by the institutional review board of Weill Cornell Medical College, which waived informed consent based on the minimal risk involved and the impracticality of obtaining consent from participants. This study followed the Strengthening the Reporting of Observational Studies in Epidemiology (STROBE) reporting guideline.

### Data and Study Population

Claims for a 20% random national sample of Medicare fee-for-service beneficiaries merged with Minimum Data Set (MDS) assessments for the period 2013 to 2019 served as the primary data sources for the study. The unit of analysis was the resident-quarter, meaning that residents had 1 observation per quarter until the end of the study period, the end of their NH long-term care stay, or death, depending on which occurred first. Characteristics of NH residents were derived from the MDS and the Master Beneficiary Summary File. The MDS is federally mandated for all NH residents and contains demographic information, diagnoses, and measures of both physical and cognitive functional status. The Master Beneficiary Summary File provided demographic characteristics, enrollment information, indicators for ADRD and other chronic conditions, and dates of death for decedents. Specifically, ADRD status was identified using diagnoses derived from the Centers for Medicare & Medicaid Chronic Conditions Warehouse classification, which is considered the criterion standard for dementia ascertainment in large administrative databases, based on validation from the Aging, Demographics, and Memory Study.^14,15,16^ The Medicare Provider Analysis and Review was used to identify ACS hospitalizations, and both the Medicare Provider Analysis and Review and Outpatient file were used to identify ED visits; the Carrier file was used to identify clinician visits.

For information on clinicians and their practices, we used the IQVIA Physician Database, the Medicare Accountable Care Organization (ACO) Provider file, and the Medicare Data on Provider Practice and Specialty. The IQVIA data included information on clinician demographic characteristics, in addition to training completed and medical schools attended for physicians. The ACO file provided information on clinicians’ ACO participation, and the Medicare Data on Provider Practice and Specialty indicated whether clinicians were physicians or advanced practitioners (APs; physician assistants and nurse practitioners), in addition to physician specialty. The LTCFocus database (Long Term Care: Facts on Care in the US) provided characteristics of all Medicare- and Medicaid-certified NHs in the US.^17^

The study population included long-stay NH residents aged 65 years or older with ADRD (eFigure 1 in Supplement 1). Long-stay residents were defined as those with stays of 100 days or longer in the same NH with no more than 10 consecutive days outside the facility.^5,18,19^ Residents in hospital-based NHs were excluded.^19^ We attributed each resident to a generalist physician (general practice, family medicine, internal medicine, physical medicine and rehabilitation, or geriatrics)^5^ or an AP in a given year based on a plurality of a beneficiary’s evaluation and management visits.^5,20^ In the case of a tie, random assignment was used.

### Exposure and Outcomes

Our exposure of interest was the annual attribution to a SNFist vs a non-SNFist clinician. SNFists were defined as physicians or APs who provided 80% or more of their evaluation and management visits to NH residents in a given year.^3,4,5^

Our outcomes included binary indicators reflecting the presence of any ACS hospitalizations and any ACS ED visits in a given quarter, including those that did or did not result in hospitalization during an individuals’ long-stay period.^18,19,21,22^ ACS conditions were defined using *International Classification of Diseases, Ninth Revision* (*ICD-9*) and *International Statistical Classification of Diseases and Related Health Problems, Tenth Revision* (*ICD-10*) codes identified by the Dartmouth Atlas.^23^ These events are considered generally, although not completely, preventable among long-stay residents with appropriate care in the NH, making them a proxy for quality of care.^24^ Hospitalizations and ED visits for ACS conditions have been used to identify opportunities for better in-place management of chronic and acute conditions in NHs, including among residents with dementia.^25,26,27^ Thus, our focus on ACS hospitalizations and ED visits is intended to capture potentially avoidable acute care that may signal quality differences in treating this high-risk population.

### Covariates

Covariates included the characteristics of residents, clinicians and their practices, and NHs. Resident characteristics included sociodemographic measures, as identified in the Master Beneficiary Summary File, such as age, sex, race and ethnicity, dual eligibility for Medicare and Medicaid, Medicare entitlement due to disability, marital status, and clinical characteristics such as baseline cognitive function scale score, activities of daily living score, antipsychotic medication use, obesity, and indicators for 75 clinical conditions and treatments (eTable 2 in Supplement 1).

Clinician characteristics included age, sex, specialty (for physicians only), whether trained in or outside the US (for physicians only), and rural location. A measure of clinician ACO participation was included to address the potential association of value-based care practice and care quality. We also included annual measures of the number of NHs each clinician visited and the percentage of residents with ADRD among all long-stay residents who received care from the clinician. Additionally, a measure of each clinician’s practice group size, defined as the number of National Provider Identifiers billing Medicare under the same primary Taxpayer Identification Number, was included.

NH characteristics included ownership type (ie, for profit vs other), affiliation with a multifacility organization, size (ie, total number of beds), occupancy rate, direct-care staff hours per resident day, the presence of any APs, the proportion of residents with stays covered by Medicare, the proportion of residents with stays covered by Medicaid, the ratio of registered nurses to all nurses, and the presence of a dementia special care unit.

### Statistical Analysis

#### Unadjusted Analysis

During the study period, we compared the baseline characteristics of residents who were ever cared for by SNFists with those who were never cared for by SNFists, using the first observation of each resident. We also compared the characteristics of SNFists vs non-SNFists, in addition to the characteristics of NHs where SNFists vs non-SNFists provided evaluation and management services, in the most recent year of the study period (2019). In unadjusted comparisons of resident, clinician, and NH characteristics, as well as outcome measures, we used *t* tests for continuous measures and χ^2^ tests for categorical measures (a 2-sided, *P* < .05 was considered statistically significant).

#### Adjusted Analysis

The study used a machine learning approach that included a doubly robust procedure incorporating a propensity score analysis with inverse probability treatment weights in a generalized estimating equation to estimate the association between the receipt of care from a SNFist and outcomes (see eMethods in Supplement 1 for details). We conducted 3 secondary analyses. First, stratified analyses were conducted for residents who received care from physicians and for those who received care from APs. Second, we conducted a sensitivity analysis without restricting the inclusion of physicians based on specialty (ie, physicians were not limited to generalists). Finally, considering death as a competing outcome, we focused on the association between SNFist care and in-place end-of-life care, building on existing evidence associating SNFists with fewer burdensome transitions at the end of life among NH decedents.^5^ We used indicators for “death without ACS hospitalization” and “death without any hospitalization” as proxy outcomes for in-place deaths, hypothesizing that SNFist care would be associated with increased odds of these outcomes. All analyses were performed from June 1, 2024, to May 3, 2025, using SAS statistical software, version 9.4 (SAS Institute Inc) and Stata/MP, version 17.0 (StataCorp LLC).

## Results

### Unadjusted Comparisons

The final sample consisted of 417 378 unique long-stay NH residents who received care from 30 286 physicians and 19 377 APs over the study period. Compared with the 174 838 residents who never received care from SNFists, the 242 540 residents who received care from SNFists were, on average, younger (mean [SD] age, 83.5 [8.7] vs 84.8 [8.5] years; *P* < .001) and less likely to be female (68.8% vs 69.8%; *P* < .001) or widowed (52.4% vs 54.8%; *P* < .001). Residents who received care from SNFists were more likely to be Black (12.6% vs 9.4%; *P* < .001), dually eligible (77.5% vs 73.1%; *P* < .001), eligible for Medicare due to disability (1.4% vs 1.1%; *P* < .001), and to receive antipsychotic medications (23.0% vs 21.0%; *P* < .001). They were more likely to have obesity (14.6% vs 12.8%; *P* < .001), anemia (60.9% vs 57.6%; *P* < .001), diabetes (43.0% vs 41.0%; *P* < .001), depressive disorder (60.5% vs 55.2%; *P* < .001), depression (57.5% vs 52.2%; *P* < .001), and hyperlipidemia (54.8% vs 52.6%; *P* < .001), but the differences were small in magnitude (Table).

**Table.** Baseline Characteristics of Nursing Home Residents With ADRD Who Ever Received Care From SNFists vs Those Who Never Received Care From SNFists^a^

Characteristic	% of Residents		*P* value^b^
	Never received care from SNFists (n = 174 838)	Ever received care from SNFists (n = 242 540)	
Age, mean (SD), y	84.8 (8.5)	83.5 (8.7)	<.001
Age group, y			
65-69	5.9	7.9	<.001
70-74	8.2	10.2	<.001
75-79	12.0	13.6	<.001
80-84	17.8	18.3	<.001
≥85	56.1	50.1	<.001
Sex			
Female	69.8	68.8	<.001
Male	30.2	31.2	
Race and ethnicity			
Black	9.4	12.6	<.001
White	85.3	83.4	
Other race or Hispanic ethnicity^c^	5.3	4.1	
Dually eligible	73.1	77.5	<.001
Disabled as reason for Medicare entitlement	1.1	1.4	<.001
Antipsychotic use	21.0	23.0	<.001
Mean (SD) CFS core	2.2 (0.8)	2.1 (0.8)	<.001
CFS score			
1 (Cognitively intact)	25.7	27.1	<.001
2 (Mildly impaired)	31.3	31.5	
3-4 (Moderately or severely impaired)	43.0	41.4	
Baseline ADL score, mean (SD)	16.2 (6.4)	16.2 (6.3)	.92
Obesity	12.8	14.6	<.001
Marital status			
Married	22.2	21.6	<.001
Widowed	54.8	52.4	
Other	23.0	26.0	
Chronic conditions^d^			
Anemia	57.6	60.9	<.001
Diabetes^e^	41.0	43.0	<.001
CHF^e^	43.5	43.6	.70
COPD^e^	26.2	26.5	.09
Cancer	9.8	9.7	.12
Hypertension^e^	86.3	87.4	<.001
Depressive disorder	55.2	60.5	<.001
Depression	52.2	57.5	<.001
Rheumatoid arthritis or osteoarthritis	53.3	54.1	<.001
Hyperlipidemia	52.6	54.8	<.001

Abbreviations: ADL, activities of daily living; ADRD, Alzheimer disease and related dementias; CFS, Cognitive Function scale; CHF, congestive heart failure; COPD, chronic obstructive pulmonary disease; SNFist, nursing home or skilled nursing facility specialist.

^a^ SNFists were defined as clinicians whose evaluation and management (E&M) visits in nursing homes (Healthcare Common Procedure Coding System codes 99304-99310, 99315, 99316, 99318) were 80% or more of all their E&M claims in a given year. Non-SNFist clinicians were defined as clinicians with at least 1 E&M visit in a nursing home but were less than 80% of all their E&M claims. The first quarterly assessment of each long-stay resident was used to describe their baseline characteristics.

^b^ We used *t* tests for continuous variables and χ^2^ tests for categorical variables to determine if differences between the residents treated by non-SNFists and residents treated by SNFists were statistically significant (2-sided *P* < .05).

^c^ Measures of race and ethnicity were taken from the Master Beneficiary Summary File (other race or Hispanic ethnicity includes Asian, North American Native, and non-White Hispanic).

^d^ Chronic conditions included the 10 most common in the study sample.

^e^ Ambulatory care–sensitive conditions.

Among all residents in the sample (N = 417 378), 49 547 (11.9%) had at least 1 ACS hospitalization during the study period, with 29 339 residents (12.1%) who received care from SNFists and 20 208 residents (11.6%) who received care from non-SNFists experiencing these events. There were 42 886 residents (10.3%) who had at least 1 ACS ED visit, including 25 193 residents (10.4%) who received care from SNFists and 17 693 residents (10.1%) who received care from non-SNFists.

The sample included 2857 physician SNFists (20.7%), 10 914 non-SNFist physicians (79.3%), 6625 AP SNFists (67.8%), and 3151 non-SNFist APs (32.2%) in 2019 (eTable 4 in Supplement 1). Compared with non-SNFist physicians (10 914), SNFist physicians (2857) were more likely to be aged 70 years or older (345 [12.1%] vs 1042 [9.5%]; *P* < .001) and female (1060 [37.1%] vs 2539 [23.3%]; *P* < .001), were less likely to be ACO participants (1008 [35.3%] vs 5624 [51.5%]; *P* < .001) and practicing in rural areas (267 [9.3%] vs 2773 [25.4%]; *P* < .001), and tended to practice at more facilities (mean [SD] number of facilities, 9.4 [8.7] for SNFists vs 6.4 [6.1] for non-SNFists). AP SNFists tended to provide care in more NHs (mean [SD] number of NHs, 8.6 [8.1] vs 7.1 [6.8]; *P* < .001) and were less likely to be affiliated with an ACO (1897 [28.6%] vs 1163 [36.9%]; *P* < .001) and to practice in a rural area (534 [8.1%] vs 635 [20.2%]; *P* < .001) compared with non-SNFist APs.

The characteristics of NHs with any residents who received care from SNFists (11 486 NHs) differed from those without any residents who received care from SNFists (2526 NHs) (eTable 5 in Supplement 1). In 2019, NHs with residents who received care from SNFists were more likely to be for profit (9256 [80.6%] vs 1650 [65.3%]; *P* < .001), part of a multifacility organization (8299 [72.3%] vs 1479 [58.6%]; *P* < .001), have a higher mean (SD) number of total beds (119.9 [61.1] vs 81.5 [40.6]; *P* < .001), have a higher mean (SD) proportion of residents covered by Medicaid (69.7% [19.6%] vs 62.0% [24.5%]; *P* < .001), have a higher mean (SD) occupancy rate (87.8% [12.0%] vs 84.0% [14.5%]; *P* < .001), and have physician extenders (8111 [70.6%] vs 1009 [39.9%]; *P* < .001), compared with NHs without residents attributed to SNFists.

### Adjusted Comparisons

Estimates from our machine learning approach using a doubly robust procedure indicated that, compared with receipt of care from a non-SNFist (physician or AP), receipt of care from a SNFist was associated with 7% lower odds of experiencing an ACS hospitalization (odds ratio [OR], 0.93; 95% CI, 0.90-0.96; *P* < .001) and 7% lower odds of having ACS ED visit (OR, 0.93; 95% CI, 0.90-0.96; *P* < .001) among residents with ADRD (Figure).

**Figure.**
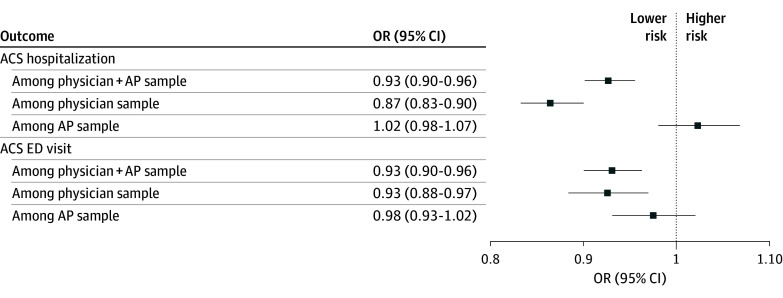
Adjusted Odds of Experiencing an Ambulatory Care–Sensitive (ACS) Hospitalization or an ACS Emergency Department (ED) Visit Among Nursing Home Residents With Alzheimer Disease and Related Dementias Who Received Care From Nursing Home or Skilled Nursing Facility Specialists (SNFists) vs Non-SNFists The estimates were obtained using a generalized estimating equation incorporating inverse probability treatment weights. The propensity scores were computed using machine learning for variable selection that accounts for nonlinearities and interactions, using the Super Learning algorithm, which builds predictions as a weighted average of predictions provided by a user given list of candidate algorithms. A detailed description of the propensity score calculation and the machine learning algorithm is provided in the eMethods in Supplement 1. AP indicates advanced practitioners; OR, odds ratio.

In secondary analyses stratified based on whether attributed clinicians were physicians or APs, receipt of care from a physician SNFist was associated with 13% lower odds of an ACS hospitalization (OR, 0.87; 95% CI, 0.83-0.90; *P* < .001) and 7% lower odds of an ACS ED visit (OR, 0.93; 95% CI, 0.88-0.97; *P* = .001) compared with receipt of care from a non-SNFist physician. Differences between residents who received care from SNFist APs and residents who received care from non-SNFist APs in the likelihood of an ACS hospitalization or an ACS ED visit were not statistically significant.

The results from the secondary analysis in which residents received care from physicians of any specialty and APs were consistent with the primary findings (ACS hospitalization OR, 0.93; 95% CI, 0.91-0.96; *P* < .001; ACS ED visit OR, 0.93; 95% CI, 0.90-0.96; *P* < .001). In our secondary analysis examining the association between SNFist care and in-place end-of-life care, we found that SNFist care was associated with 7% higher odds of death without an ACS hospitalization (OR, 1.07; 95% CI, 1.05-1.08; *P* < .001) and 9% higher odds of death without any hospitalization (OR, 1.09; 95% CI, 1.07-1.11; *P* < .001) compared with residents who did not receive SNFist care.

## Discussion

Nearly half of all hospitalizations and ED visits by NH residents are related to ACS conditions,^28,29,30^ which should be largely preventable.^31,32^ In this nationwide cohort study, we found that receipt of care from SNFists, including both physicians and APs, may lead to higher-quality care for long-stay residents with ADRD based on a lower likelihood of experiencing these events. This association was also observed in comparisons of SNFist physicians with non-SNFist physicians, but not in comparisons of SNFist APs with non-SNFist APs. These findings suggest that NH-focused care by clinicians may improve the quality of care for residents with ADRD. Although the lower odds of hospitalization and ED visits for ACS conditions associated with SNFists were relatively modest in magnitude, they do suggest improvements in the quality of care. Given the elevated risk of these events for NH residents with ADRD compared with other residents, even small reductions are important.

Our findings add to a growing body of evidence suggesting that NH use of SNFists may improve the quality of care for residents. Previous research has found an association of care by SNFists with lower rehospitalization rates and timely discharge to the community for postacute patients, as well as lower rates of antipsychotic medication prescribing and indwelling bladder catheter use.^3,4,20^ Additionally, a 2024 study found an association between receipt of care from SNFists and reductions in burdensome transitions at the end of life among NH residents.^5^

Better outcomes observed among residents associated with care provided by SNFists in our analyses may have occurred through multiple mechanisms. These include SNFists’ development of specialized knowledge of NH regulations and medication guidelines for residents with ADRD, along with an in-depth understanding of care for patients with clinically complex conditions.^33,34,35^ SNFists may also be more experienced than other clinicians in addressing the behavioral and psychological symptoms that frequently accompany ADRD. Additionally, NH-focused practice may enhance communication with direct-care staff and administration in the facility and may help build long-term relationships with residents and their families.

The prevalence of SNFists has increased rapidly, and these clinicians now provide care in more than half of all US NHs.^3^ The increase of SNFists signifies a growing acknowledgment of the importance of care tailored to specific settings, similar to the established role of hospitalists in inpatient care.^5^ However, challenges such as limited exposure to NH care during medical training,^5^ financial disincentives associated with practicing in NHs vs acute settings,^2,36^ and the absence of clearly defined specialization pathways may hamper the development of clinicians pursuing practice in NHs.

Despite the benefits of SNFist care suggested by our results, there may be differences in the quality of care based on the practice arrangements of these clinicians. For example, continuity with primary care clinicians has been associated with better outcomes for NH residents^19^ and is particularly important for individuals with ADRD who rely on caregivers and clinicians to monitor symptoms, maintain medical histories, and track treatment response over time. Although all SNFists primarily practice in NHs, some may focus their practice in a small number of facilities where they regularly provide care to the same residents, whereas other SNFists may provide care across a large number of NHs and do not develop familiarly with residents. Similarly, continuity of care may differ between short-stay residents admitted for postacute care and long-stay residents who reside in the facility for extended periods.^37,38^ Further research is needed to explore the effects of diverse SNFist practice patterns on care outcomes.

Findings from our secondary analysis of in-place end-of-life care suggest that the use of SNFists may contribute to a shift toward in-place deaths, reducing unnecessary hospitalizations, an indicator of improved quality of care among long-stay residents with ADRD.^39,40,41^ Our findings also align with prior research suggesting that SNFist care may reduce burdensome transitions at the end of life.^5^

### Limitations

Our study has limitations. First, we did not have data for residents enrolled in Medicare Advantage during the study period, limiting our findings to residents enrolled in traditional Medicare. Second, although we followed the attribution logic developed by the Centers for Medicare & Medicaid to attribute residents to clinicians that has been widely used in previous research,^5,19,21,36,42,43,44^ the logic may not have assigned residents to the clinician primarily overseeing their care in some cases. However, any mismeasurement would have biased our results toward no effect. Third, our attribution logic may not fully capture team-based care or other types of value-based care practice, which is not necessarily reflected in evaluation and management billings.^45^ For example, in team-based care models, APs often work in conjunction with physicians and other health care professionals in NHs. This may have contributed to the insignificant findings in our comparisons of SNFist APs with non-SNFist APs. Similarly, under value-based payment arrangements such as ACOs, some evaluation and management visits may not be fully captured in claims, potentially leading to an underestimation of care provision. For example, an ACO may have had clinicians periodically make unbilled visits to their attributed beneficiaries in NHs to reduce the likelihood of hospitalization. To address this concern, our analytic models included an indicator for clinicians’ ACO participation, although this would not capture the influence of other value-based programs. Fourth, we used proxy outcomes for in-place death. There is a possibility that in-hospital deaths were classified as nonhospital deaths due to discrepancies in the timing of hospital admissions and discharges relative to death, and vice versa. However, they are unlikely to bias our findings in a specific direction and remain a valuable proxy for assessing end-of-life care patterns. Further research incorporating detailed data on care preferences and goal-concordant care, hospice use, and place of death would be valuable in elucidating the effect of SNFists on the quality end-of-life care. Although we used a rigorous machine learning approach with a rich set of resident, clinician, and facility characteristics, it is possible that unobserved factors correlated with both SNFist care and resident outcomes influenced our estimates. Therefore, our findings should not be interpreted as causal effects.

## Conclusions

The findings of this national cohort study suggest that SNFists were associated with improved quality of care for long-stay NH residents with ADRD based on lower odds of hospitalization and ED visits for ACS conditions.

## Notes

Supplement 1. **eTable 1.** Abbreviations Used in the Study **eTable 2.** Resident Chronic Conditions and Treatments Used in Adjusted Estimates **eFigure 1.** Selection of the Study Sample
eMethods.
**eTable 3.** Machine Learning (Super Learning Algorithm) Weights **eFigure 2.** Standardized Differences for Resident-level Baseline Covariates Comparing Treated to Untreated Individuals in the Original and the Weighted Sample **eTable 4.** Characteristics of SNFists vs. Non-SNFists, 2019 **eTable 5.** Characteristics of Nursing Homes With Residents Attributed to SNFists vs. Those Without Residents Attributed to SNFists, 2019
eReferences.

Supplement 2.
Data Sharing Statement
