# Supplementary material for: Clinicians Who Practice Primarily in Nursing Homes and the Quality of Care for Residents With Alzheimer Disease and Related Dementias
Source: JAMA Health Forum. 2025 Aug 15;6(8):e252465. doi: 10.1001/jamahealthforum.2025.2465 (PMC12357189; doi:10.1001/jamahealthforum.2025.2465)
Supplement: Supplement 1. — eTable 1. Abbreviations Used in the Study eTable 2. Resident Chronic Conditions and Treatments Used in Adjusted Estimates eFigure 1. Selection of the Study Sample eMethods. eTable 3. Machine Learning (Super Learning Algorithm) Weights eFigure 2. Standardized Differences for Resident-level Baseline Covariates Comparing Treated to Untreated Individuals in the Original and the Weighted Sample eTable 4. Characteristics of SNFists vs. Non-SNFists, 2019 eTable 5. Characteristics of Nursing Homes With Residents Attributed to SNFists vs. Those Without Residents Attributed to SNFists, 2019 eReferences. [file jamahealthforum-e252465-s001.pdf]

Supplemental Online Content

Yun H, Unruh MA, Qian Y, Zhang Y, Jung HY. Clinicians who practice primarily in nursing homes and the quality of care for residents with Alzheimer disease and related dementias. *JAMA Health Forum*. 2025;6(8):e252465.
doi:10.1001/jamahealthforum.2025.2465

eTable 1. Abbreviations Used in the Study

eTable 2. Resident Chronic Conditions and Treatments Used in Adjusted Estimates

eFigure 1. Selection of the Study Sample

eMethods.

eTable 3. Machine Learning (Super Learning Algorithm) Weights

eFigure 2. Standardized Differences for Resident-level Baseline Covariates Comparing Treated to Untreated Individuals in the Original and the Weighted Sample

eTable 4. Characteristics of SNFists vs. Non-SNFists, 2019

eTable 5. Characteristics of Nursing Homes With Residents Attributed to SNFists vs. Those Without Residents Attributed to SNFists, 2019

eReferences.

This supplemental material has been provided by the authors to give readers additional information about their work.

© 2025 Yun H et al. *JAMA Health Forum*.

eTable 1. Abbreviations Used in the Study

Abbreviations	Definitions
NH	Nursing home
SNFist	Nursing home or skilled nursing facility specialist (i.e. clinicians providing care almost exclusively in nursing homes)
ADRD	Alzheimer disease and related dementias
ED	Emergency department
ACS	Ambulatory care sensitive
MDS	Minimum Data Set
MBSF	Master Beneficiary Summary File
ACO	Accountable Care Organization
MD-PPAS	Medicare Data on Provider Practice and Specialty
AP	Advanced practitioner (physician assistants and nurse practitioners)
PA	Physician assistant
NP	Nurse practitioners
LTCFocus	Long Term Care: Facts on Care in the U.S.
E&M	Evaluation and Management
CFS	Cognitive function scale
ADL	Activities of daily living
CHF	Congestive heart failure
COPD	Chronic obstructive pulmonary disease
RN	Registered nurses
GEE	Generalized estimating equation
IPTW	Inverse probability of treatment weighting
SD	Standard deviation
OR	Odds ratio
CI	Confidence interval
CV	Cross-validated

© 2025 Yun H et al. *JAMA Health Forum*.

eTable 2. Resident Chronic Conditions and Treatments Used in Adjusted Estimates[a]

Chronic Conditions
1 Acute Myocardial Infarction
2 Anemia
3 Asthma
4 Atrial Fibrillation
5 Breast cancer
6 Colorectal cancer
7 Endometrial cancer
8 Lung cancer
9 Prostate cancer
10 Cataract
11 Heart failure
12 Chronic kidney disease
13 Chronic Obstructive Pulmonary Disease
14 Depression
15 Diabetes
16 Glaucoma
17 Hip fracture
18 Hyperlipidemia
19 Hyperplasia
20 Hypertrophy
21 Hypothyroidism
22 Ischemic Heart Disease
23 Osteoporosis
24 Rheumatoid arthritis, Osteoarthritis
25 Stroke / Transient Ischemic Attack
26 ADHD and Other Conduct Disorders
27 Alcohol Use Disorder
28 Anxiety
29 Autism
30 Bipolar Disorder
31 Brain Injury
32 Cerebral Palsy
33 Cystic Fibrosis
34 TO10 Depression
35 Drug Use Disorder
36 Epilepsy
37 Chronic Pain, Fatigue, and Fibromyalgia
38 Hearing Impaired
39 Viral Hepatitis
40 HIV/AIDS
41 Intellectual Disabilities
42 Learning Disabilities
43 Leukemias and Lymphomas
44 Liver Disease, Cirrhosis & Oth Liver Cond (excl Hepatitis)
45 Migraine and other Chronic Headache
46 Mobility Impairments
47 Multiple Sclerosis and Transverse Myelitis
48 Muscular Dystrophy

© 2025 Yun H et al. *JAMA Health Forum*.

49	Other Developmental Delays
50	Overarching OUD Disorder (Any of the 3 Sub-Indicators)
51	Diagnosis and Procedure Basis for OUD
52	Opioid-Related Hospitalization or Emergency Department
53	Use of Medication-Assisted Treatment (MAT)
54	Personality Disorders
55	PTSD
56	Peripheral Vascular Disease
57	Schizophrenia
58	Schizophrenia and Other Psychotic Disorders
59	Spina Bifida and Other Congenital Anomalies
60	Spinal Cord Injury
61	Tobacco Use Disorders
62	Pressure Ulcers and Chronic Ulcers
63	Sensory Blindness and Visual Impairment
64	Antianxiety medication use
65	Antidepressant medication use
66	Hypnotic medication use
67	Post parenteral/IV feeding
68	Post feeding tube
69	Post mechanically altered diet
70	Intravenous medication post-admit
71	Transfusion post-admit
72	Active diagnosis for anxiety disorder
73	Active diagnosis for manic depression
74	Active diagnosis for psychotic
75	Diagnosis for schizophrenia

[a] Chronic conditions were identified from the Master Beneficiary Summary File chronic conditions segments. Treatments were identified from the Minimum Data Set.

© 2025 Yun H et al. *JAMA Health Forum*.

eFigure 1. Selection of the Study Sample

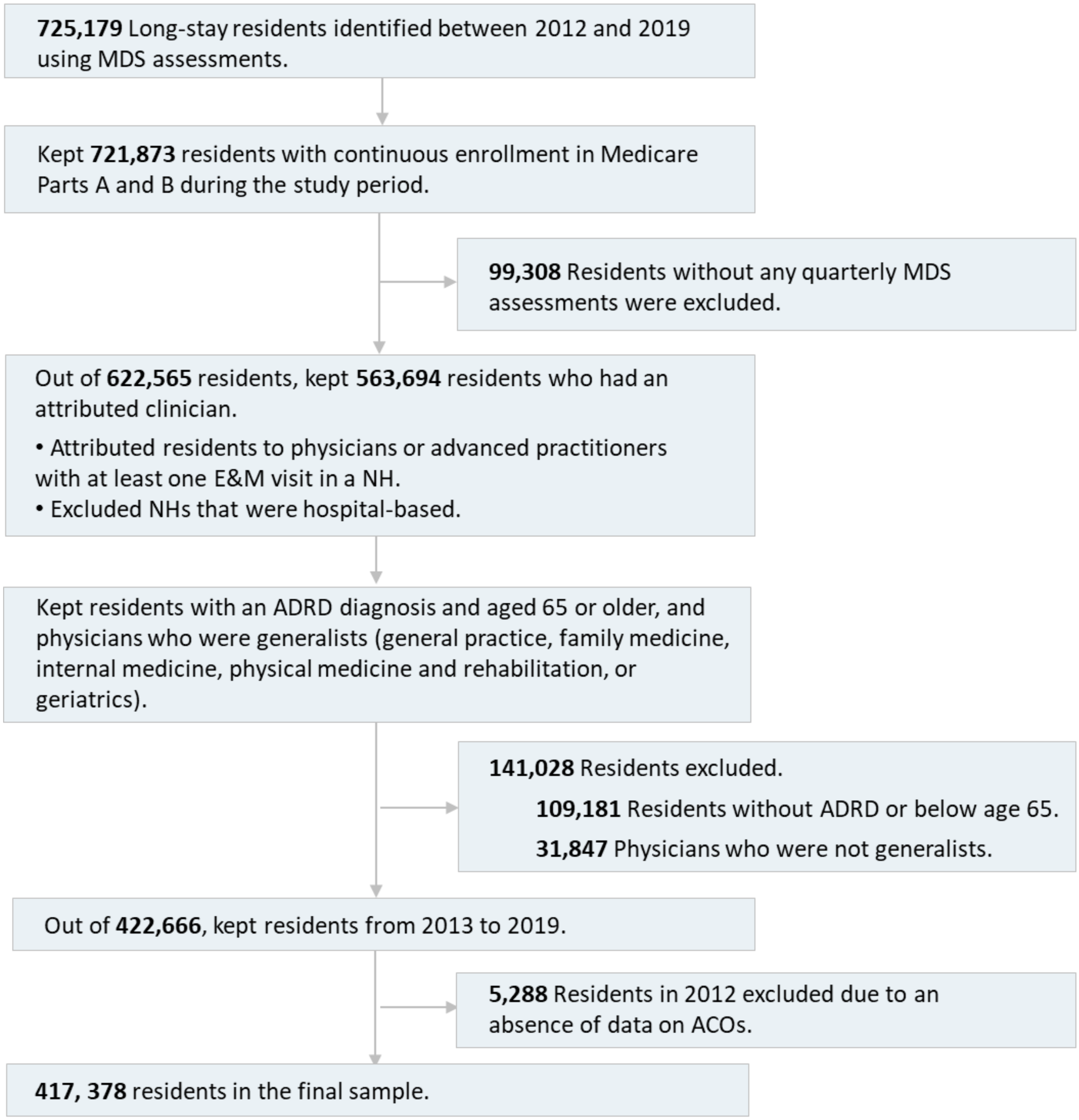

Abbreviations: MDS, Minimum Data Set. NH, nursing home. E&M, evaluation and management. ADRD, Alzheimer Disease and Related Dementias.

© 2025 Yun H et al. *JAMA Health Forum*.

eMethods.

The study utilized a double-robust procedure using propensity score analysis with inverse probability weights in a generalized estimating equation (GEE) approach to estimate the relationship between attribution to a SNFist (vs. a non-SNFist) and outcomes of nursing home residents with Alzheimer disease and related dementias (ADRD). The propensity score is the probability of a clinician being a SNFist given an observed set of baseline clinician characteristics. Our estimators of the propensity score relied on machine learning for variable selection, which also automatically accounted for non-linearities and interactions in the model, using the Super Learning algorithm.[1,2] Specifically, we fit the propensity to be a SNFist as a function of clinician characteristics using Super Learning, which built predictions as a weighted average of predictions provided by a user given list of candidate algorithms, including logistic regression (*logit*), LASSO (*lasso*), classification/regression tree with cross validated selection of meta parameters (*cvcart*), and random forest (*rfoob*), where the average weights were chosen to minimize the out-of-sample prediction error. The weights that were given to each algorithm in the propensity score prediction are listed in the **Supplement, eTable 3**.

We then used inverse probability treatment weighting (IPTW) to assign weights to the clinicians. The IPTW was $1/P_i$ for clinicians who were SNFists in a given year and $1/(1-P_i)$ for clinicians who were not SNFists in a given year (where P_i is the propensity score of clinician *i*). The IPTW analysis was used to reduce reliance of our method on the correct specification of the outcome model (GEE), and to address treatment-selection bias as well as potential confounders inherent in observational studies. Once we applied the weights to residents attributed to clinicians, the standardized differences between those treated by SNFists vs. non-SNFists were reduced and fell below the 10% threshold widely used in studies (**Supplement, eFigure 2**).[3]

Lastly, we used a binomial GEE approach incorporating the IPTW with the logit link function for outcomes. All GEE regression analyses were conducted at the resident-quarter level, controlling for resident, clinician, and nursing home characteristics. An autoregressive first-order (AR(1)) correlation structure was specified in the GEE procedure. Standard errors were adjusted for clustering at the nursing home level.

© 2025 Yun H et al. *JAMA Health Forum*.

eTable 3. Machine Learning (Super Learning Algorithm) Weights

Learner	Coefficient	CV Risk
Logit	0.05723	0.17559
Lasso	0.00000	0.18518
Cvcart	0.05102	0.17162
Rfoob	0.89175	0.16222

Abbreviations: CV, cross-validated.

© 2025 Yun H et al. *JAMA Health Forum*.

eFigure 2. Standardized Differences for Resident-level Baseline Covariates Comparing Treated to Untreated Individuals in the Original and the Weighted Sample

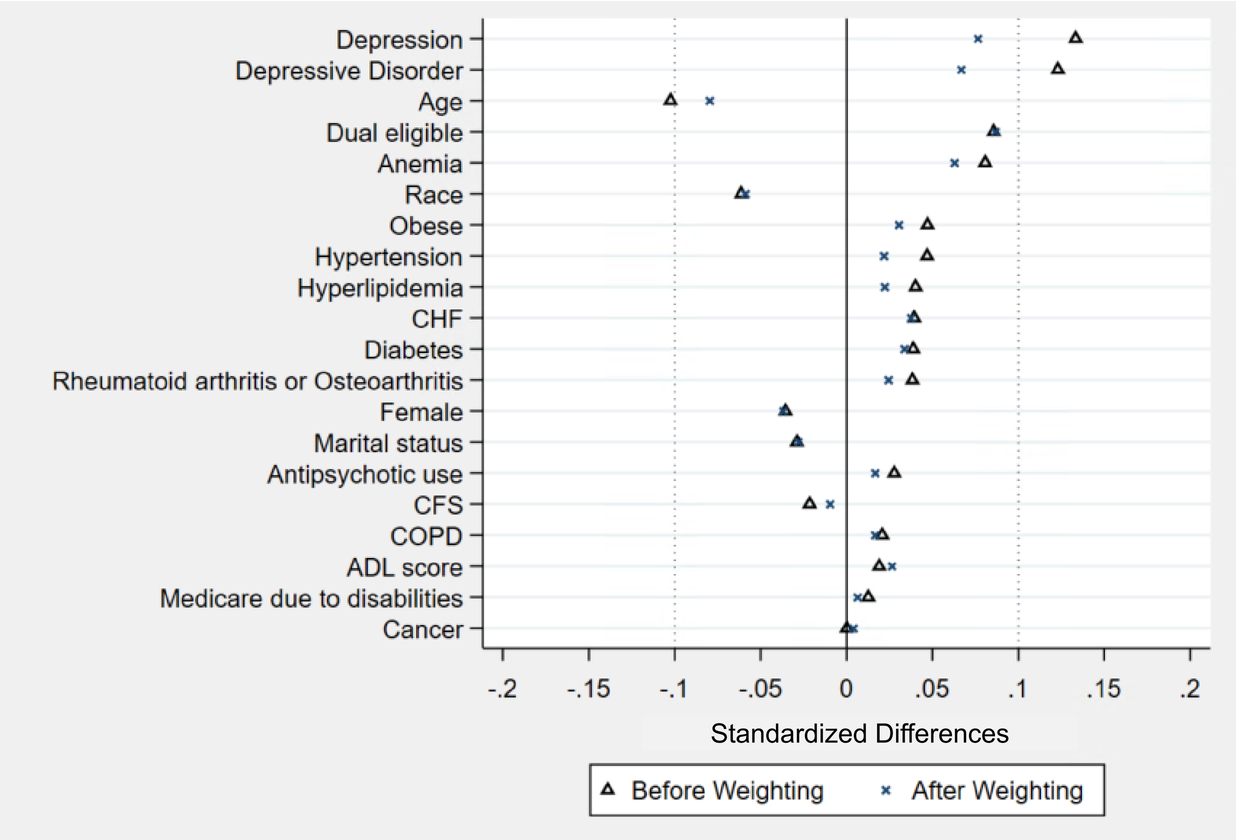

© 2025 Yun H et al. *JAMA Health Forum*.

eTable 4. Characteristics of SNFists vs. Non-SNFists, 2019

	Physicians			Advanced Practitioners (APs)		
	SNFists[a]	Non-SNFists[a]	P value	SNFists[a]	Non-SNFists[a]	P value
**Total N (%)**	2,857(20.7)	10,914(79.3)	NA	6,625(67.8)	3,151(32.2)	NA
**Top 5 specialties, N (%)**						
Family Medicine	997 (34.9)	4,902 (44.9)	< .001	NA	NA	NA
General Practice	135 (4.7)	232 (2.1)				
Geriatric Medicine	236 (8.3)	335 (3.1)				
Internal Medicine	1,237 (43.3)	5,298 (48.5)				
Physical Medicine and Rehabilitation	252 (8.8)	147 (1.3)				
**Age Categories, N (%)**						
Mean (SD)	54.5(12.4)	55.9(10.9)	< .001	45.3(10.7)	45.4(10.7)	.91
<30	4 (0.1)	8 (0.1)	< .001	242 (3.7)	121 (3.8)	.72
[30, 39]	385 (13.5)	933 (8.5)		2045 (30.9)	981 (31.1)	
[40, 49]	673 (23.6)	2175 (19.9)		2101 (31.7)	972 (30.8)	
[50, 59]	719 (25.2)	3316 (30.4)		1433 (21.6)	714 (22.7)	
[60, 69]	731 (25.6)	3440 (31.5)		733 (11.1)	335 (10.6)	
≥70	345 (12.1)	1042 (9.5)		71 (1.1)	28 (0.9)	
**Female, N (%)**	1060 (37.1)	2539 (23.3)	< .001	5837 (88.1)	2681 (85.1)	< .001
**Foreign Trained, N (%)**	1066 (37.3)	4135 (37.9)	.25	NA	NA	NA
**Participation in an ACO, N (%)**	1008 (35.3)	5624 (51.5)	< .001	1897 (28.6)	1163 (36.9)	< .001
**Practice Size[b], N (%)**						
Mean (SD)	148.9(437.9)	152.4(393.5)	.69	151.7(412.2)	147.4(401.0)	.63
Solo	632 (22.1)	3287 (30.1)	< .001	271 (4.1)	163 (5.2)	< .001
[2, 9]	486 (17.0)	2644 (24.2)		1386 (20.9)	921 (29.2)	
[10, 49]	791 (27.7)	1579 (14.5)		2293 (34.6)	925 (29.4)	
[50, 99]	282 (9.9)	687 (6.3)		914 (13.8)	356 (11.3)	
[100+]	666 (23.3)	2717 (24.9)		1761 (26.6)	786 (24.9)	
**Rural, N (%)**	267 (9.3)	2773 (25.4)	< .001	534 (8.1)	635 (20.2)	< .001
**Number of nursing homes where**	9.4(8.7)	6.4(6.1)	< .001	8.6(8.1)	7.1(6.8)	< .001

© 2025 Yun H et al. *JAMA Health Forum*.

**clinicians provided care**						

Abbreviations: ACO, Accountable Care Organization.

a. SNFists were defined as clinicians whose evaluation and management (E&M) visits in nursing homes (Healthcare Common Procedure Coding System [HCPCS] Codes 99304-99310, 99315, 99316, 99318) were ≥ 80% of all their E&M claims in a given year. Non-SNFist clinicians were defined as clinicians with at least one E&M visit in a nursing home but were less than 80% of all their E&M claims. The table is based on data from 2019.
b. Practice size was defined as the number of unique National Provider Identifiers billing Medicare under the same primary Taxpayer Identification Number.
We used t-tests for continuous variables and chi-square tests for categorical variables to determine if differences between SNFist and non-SNFist physicians, as well as differences between SNFist and non-SNFist APs, were statistically significant (2-sided, $P < 0.05$).

© 2025 Yun H et al. *JAMA Health Forum*.

eTable 5. Characteristics of Nursing Homes With Residents Attributed to SNFists vs. Those Without Residents Attributed to SNFists, 2019

	NHs with SNFists[a]	NHs without SNFists[a]	P value[b]
**Total N (%)**	11486 (82.0)	2526 (18.0)	
**For-profit (%)**	9256 (80.6)	1650 (65.3)	< .001
**Multi-Facility Organization Affiliation (%)**	8299 (72.3)	1479 (58.6)	< .001
**Total beds, Mean (SD)**	119.9 (61.1)	81.5 (40.6)	< .001
**% Medicare, Mean (SD)**	18.6 (12.5)	15.4 (13.0)	< .001
**% Medicaid, Mean (SD)**	69.7 (19.6)	62.0 (24.5)	< .001
**Occupancy rate, Mean (SD)**	87.8 (12.0)	84.0 (14.5)	< .001
**Total direct care hours/resident/day, Mean (SD)**	3.9(1.0)	3.9 (1.2)	.61
**Ratio of RN to all nurses, Mean (SD)**	0.4 (0.2)	0.4 (0.2)	.002
**Physician extender: facility has NP or PA (%)**	8111 (70.6)	1009 (39.9)	< .001
**Alzheimer's disease special care unit (%)**	2715 (23.6)	470 (18.6)	< .001

Abbreviations: NH, Nursing home. NP, Nurse practitioner. PA, Physician assistance. RN, Registered Nurse.

a. SNFists were defined as clinicians whose evaluation and management (E&M) visits in nursing homes (Healthcare Common Procedure Coding System Codes 99304-99310, 99315, 99316, 99318) were ≥ 80% of all their E&M claims in a given year. Non-SNFist clinicians were defined as clinicians with at least one E&M visit in a nursing home, but less than 80% of all their E&M claims.
b. We used t-tests for continuous variables and chi-square tests for categorical variables to examine whether differences were statistically significant (2-sided, $P < 0.05$).

eReferences.

1. van der Laan MJ, Polley EC, Hubbard AE. Super learner. *Stat Appl Genet Mol Biol*. 2007;6:Article25. doi:10.2202/1544-6115.1309
2. Keil AP. Super learning in the SAS system. *ArXiv*. 2018;abs/1805.08058
3. Chesnaye NC, Stel VS, Tripepi G, et al. An introduction to inverse probability of treatment weighting in observational research. *Clin Kidney J*. Jan 2022;15(1):14-20. doi:10.1093/ckj/sfab158

© 2025 Yun H et al. *JAMA Health Forum*.
