# Supplementary material for: Clinicians Who Practice Primarily in Nursing Homes and the Quality of Care for Residents With Alzheimer Disease and Related Dementias
Source: JAMA Health Forum. 2025 Aug 15;6(8):e252465. doi: 10.1001/jamahealthforum.2025.2465 (PMC12357189; doi:10.1001/jamahealthforum.2025.2465)
Supplement: Supplement 2. — Data Sharing Statement [file jamahealthforum-e252465-s002.pdf]

# Data Sharing Statement

Yun. Clinicians Who Practice Primarily in Nursing Homes and the Quality of Care for Residents With Alzheimer Disease and Related Dementias. *JAMA Health Forum*. Published August 15, 2025. doi:10.1001/jamahealthforum.2025.2465

**Data**
**Data available:** No

**Additional Information**
**Explanation for why data not available:** Our data use agreement with the Centers for Medicare and Medicaid Services does not allow us to share the data.
